# Considerations for Tissue Expansion in the Management of Massive Dermatofibrosarcoma Protuberans of the Head and Neck

**Published:** 2013-12-27

**Authors:** Austin M. Badeau, Mark Granick, Frederic W.-B. Deleyiannis

**Affiliations:** ^a^University of Colorado School of Medicine, MSIV, Aurora; ^b^Division of Plastic Surgery, Department of Surgery, Rutgers New Jersey Medical School, Newark; ^c^Division of Plastic Surgery, Departments of Surgery and Otolaryngology, University of Colorado Hospital & Children's Hospital Colorado, Aurora

**Keywords:** tissue expansion, dermatofibrosarcoma protuberans, connective tissue tumor, aggressive fibromatosis, head and neck reconstruction

## Abstract

**Objective:** Dermatofibrosarcoma protuberans (DFSP) is a locally invasive soft tissue tumor with rare malignant potential, but a high tendency for local recurrence. We present 2 cases of DFSP involving the head and neck requiring tissue expansion for reconstruction. **Methods:** A retrospective review of the medical records for 2 patients with DFSP was performed. Data concerning the operative approaches, reconstructive strategies, details of tissue expansion, and outcomes were collected. **Results:** Case 1: A 4-year-old child with a large DFSP infiltrating her entire anterolateral neck and shoulder. Case 2: A 34-year old woman with a large DFSP involving the scalp overlaying the anterior-frontal scalp. Both patients underwent successful tumor resection and reconstruction; however, the timing of tissue expander placement and final reconstruction differed. **Conclusions:** These cases present the challenges of soft tissue reconstruction of the head and neck following tumor extirpation. In addition, we discuss the important considerations for tissue expansion prior to tumor resection versus after tumor resection and free flap reconstruction.

Dermatofibrosarcoma protuberans (DFSP) is a locally invasive soft tissue tumor with rare malignant potential.^1^ DFSP may increase in size over a period of months to years producing large protuberant nodules. Despite its large size, the tumor is rarely fixed to underlying structures but is frequently complicated by local recurrence.^2^

We present 2 cases of DFSP involving the head and neck. Case 1: A 4-year-old child with a DFSP infiltrating her entire anterolateral neck and shoulder (Fig 1A). Case 2: A 34-year-old woman with a DFSP involving the anterior-frontal scalp (Fig 2A). Following tumor resection, reconstruction proceeded with appropriate soft tissue coverage as described in detail given later. Reconstruction in the 2 cases differed in that tissue expanders were placed 3 months prior to tumor resection in case 1, while tissue expansion occurred after tumor resection in case 2. These cases highlight the challenges of DFSP resection and soft tissue reconstruction of the head and neck following large tumor extirpation. In addition, we discuss the indications for tissue expansion before tumor resection versus after tumor resection.

## METHODS

Two patients who had radical DFSP resections and subsequent reconstructions with tissue expansion were identified. A retrospective review of their medical records was performed. Data concerning the operative approaches, reconstructive strategies, and outcomes were collected.

## RESULTS

### Case 1 

**Figure 1:**
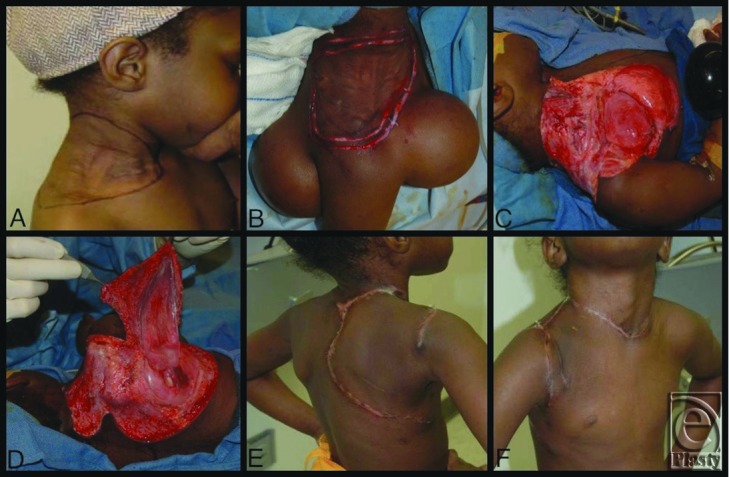
Case 1, Panel A: Palpable margins of DFSP marked; Panel B: Tissue expanders fully inflated with the initial 5 mm circumferential excision; Panel C: DFSP excised with deltopectoral flap raised; Panel D: Scapular flap raised; Panel E: Posterior view 1 month after excision and reconstruction; Panel F: Anterior view.

A 4-year-old child presented with a 2-year history of an indurated skin lesion on the right side of her neck and shoulder, which was biopsied and found to be a DFSP (Fig 1A).

The patient was brought to the operating room, where 2 tissue expanders were placed; one in the anterior chest to expand a deltopectoral flap and a second in the back to expand a parascapular flap. Tissue expansion occurred for 3 months, leading up to tumor extirpation (Fig 1B).

The primary surgical resection was done first as an extensive biopsy to determine true peripheral margins.^3^^,^^4^ A mark was made around the edge of the palpable tumor. A second line was made 1 cm beyond this, and subsequently a 5-mm circumferential excision was made beyond the 1-cm margin. The tissue was evaluated in a standard Moh fashion. Any positive margins were then re-resected and sent for repeat frozen section. Using this technique, the peripheral margins were cleared after 7 hours. The margins were then primarily closed to the central tumor mass.

Three days later, the patient was subsequently taken to surgery for resection of the central tumor mass and reconstruction of the defect. The defect after obtaining negative frozen sections of the deep cervical fascia was 16 cm × 16 cm. Reconstruction was then accomplished using the expanded deltopectoral flap (Fig 1C) and parascapular flap (Fig 1D). The patient healed well with good cosmetic result (Fig 1E/F). Unfortunately, the permanent deep margins of the central tumor mass were found to be positive. Instead of additional surgery, the family chose to be followed with serial examinations and magnetic resonance imaging scans. At last follow-up at 2 years from surgery, there was no clinical or radiologic evidence of recurrence.

### Case 2

**Figure 2:**
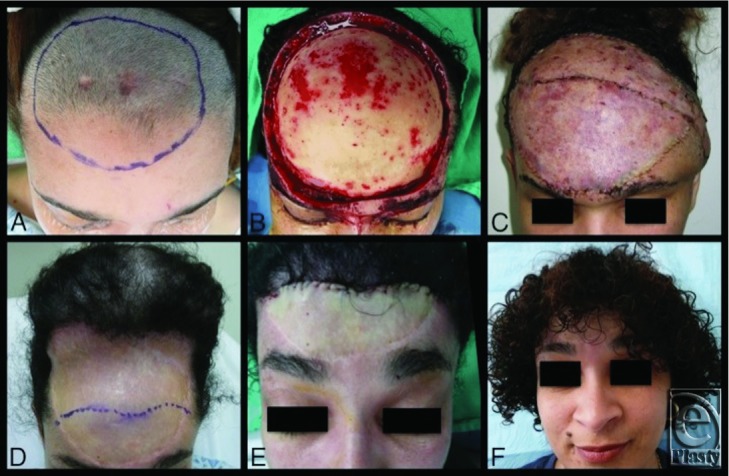
Case 2, Panel A: Palpable margins of DFSP marked; Panel B: Defect after radical excision; Panel C: Reconstruction with latissimus free flap and skin graft; Panel D: Tissue expansion for scalp reconstruction; Panel E: Restoration of anterior hairline with advancement of expanded scalp flap; Panel F: Anterior view.

A 34-year-old woman with a prior history of DFSP presented with a recurrence on her scalp. The area, which had previously been excised, demonstrated a cluster of small red macular lesions on her anterior frontal scalp (Fig 2A). After wide (5-cm margins) excision, permanent sections demonstrated positive deep and peripheral margins so a radical excision was planned. The patient was brought to the operating room, where radical excision of the tumor was performed leaving a 20 cm × 14 cm defect of her anterior scalp and forehead with exposed skull (Fig 2B). A latissimus free flap and split-thickness skin graft were harvested to provide coverage of the defect (Fig 2C).

Three months later, a tissue expander was placed under the native posterior scalp (Fig 2D). Tissue expansion took place for 3 months after which the expander was removed; the skin graft overlying the posterior aspect of the latissimus flap was excised (ie, the reconstructed anterior scalp), and the expanded scalp flap was advanced forward to restore the natural hairline with good cosmetic result (Fig 2E/F).

## DISCUSSION

DFSP demonstrates recurrence rates between 26% and 60% with conservative margins.^5^ It is advocated that margins be between 2 and 3.5 cm to achieve negative margins for adequate local control. Following these guidelines, a review of the literature reflects recurrence rates still averaging around 8.8%, leading some authors to advocate for radical tumor excision (≥5-cm margins).^5^ Recurrence rates can be especially high (50%-75%) in the head and neck, likely reflecting the reluctance to take wide margins for cosmetic and functional reasons.^5^^-^7

With Moh's micrographical surgery, recurrence rates have been reported as low as 1.5%.^5^ Some authors consider Moh's micrographical surgery as the treatment of choice in particularly anatomically challenging areas such as the head or neck^8^ and in the treatment of children.^9^

The great challenge in the 2 described cases was reconstruction of the large defect created from tumor extirpation. In both cases, reconstruction with tissue expansion was performed. However, the surgical timing of tissue expansion differed. In case 1, tissue expansion began 3 months prior to tumor resection, allowing expander explant, tumor resection, and reconstruction to occur during a single operation. In case 2, the tumor was immediately resected, and the defect was then covered by a latissimus free flap and skin graft. Six months later, a tissue-expanded scalp flap was used to restore optimal appearance by reestablishing the anterior hairline.

The question of whether to perform tissue expansion pre- versus posttumor resection is a challenging decision. The advantage of placing tissue expanders prior to tumor resection is that single-phase reconstruction can simultaneously be performed with tumor extirpation, avoiding the need for a staged approach involving the harvest of a flap or skin graft. Of course, the disadvantage entails the increased risk for tumor growth, metastasis during the period of tissue expansion, and/or the possibility of positive margins on permanent pathologic analysis. The risk of undetected tumor at a distance from the primary site (ie, site where the tumor is visible) is also a deterrent for placing tissue expanders prior to tumor resection. Tumor excision with clear margins could extend into the expanded area.

Metastasis with DFSP is rare. In a review of 913 DFSP cases, Rutgers et al^10^ found 11 patients (1%) with regional lymph node metastasis and 37 patients (4%) with visceral metastasis. The most common site of hematogenous metastasis was the lung (92%). Metastatic disease carries a very poor prognosis with a mean survival time of 14 months.^10^ The probability of metastasis is low but real. So is there a way of risk stratifying patients to predict those who will develop metastatic disease? Several studies^10^^-^12 have shown that recurrent disease, patient age more than 50, and the DFSP-fibrosarcomatous (FS) subtype are all associated with worse outcome and higher incidence of local invasion/metastasis.

Of these risk factors, the DFSP-FS subtype demonstrates the most consistent evidence of increased risk for local recurrence, metastasis, and poor outcome. This subtype tends to demonstrate a high degree of cellularity and mitotic rate. Bowne et al^12^ report a 5-year recurrence free survival rate for “classic” DFSP and “aggressive” DFSP-FS to be 81% and 28%, respectively.

While considering the timing of tissue expansion, the reconstructive surgeon should ask himself or herself 2 questions. First, can the deep and peripheral margins be definitively cleared prior to reconstruction with expanded flaps? Second, will tissue expansion eliminate the need for a flap? Defects that cross 2 different aesthetic units of the head and neck make reconstruction with tissue expansion alone very problematic. This scenario is demonstrated in case 2, in which the defect involved significant portions of both the scalp and forehead. Complete coverage of the defect with an expanded scalp flap would significantly lower the hairline yielding an unacceptable cosmetic result. This case required both tissue expansion and a free flap with skin grafting to preserve the separate aesthetic units of the face while covering the defect. On the contrary, case 1 demonstrates a scenario for which tissue expansion allowed for complete reconstruction of the defect, as there was plenty of adjacent tissue to replace “like with like.” However, note that even though the expanders were placed in sites quite distant from the visible tumor, the positive margins extended nearly to the sites of the expanded flap. As a general reconstructive rule, the defect should be determined before reconstruction is planned/initiated. If expansion is to be done before tumor resection, the expanders should be placed at a distance that is well beyond the sites of possible, positive margins. With a DFSP, this may not be possible in some (possibly the majority of) cases, given the extensive, infiltrative nature of the tumor.

Ultimately, the decision of whether to perform tissue expansion pre- versus posttumor resection should be made with the patient, conservatively weighing the probability of local tumor growth/metastasis and positive margins against the need for a staged reconstruction.
